# Bacteria and Competing Herbivores Weaken Top–Down and Bottom–Up Aphid Suppression

**DOI:** 10.3389/fpls.2018.01239

**Published:** 2018-09-03

**Authors:** Carmen K. Blubaugh, Lynne Carpenter-Boggs, John P. Reganold, Robert N. Schaeffer, William E. Snyder

**Affiliations:** ^1^Department of Plant and Environmental Sciences, Clemson University, Clemson, SC, United States; ^2^Department of Entomology, Washington State University, Pullman, WA, United States; ^3^Department of Crop and Soil Sciences, Washington State University, Pullman, WA, United States

**Keywords:** tritrophic interactions, *Brassica oleracea*, Bacillus spp., Pseudomonas spp., Brevicoryne brassicae, growth-defense tradeoff

## Abstract

Herbivore suppression is mediated by both plant defenses and predators. In turn, plant defenses are impacted by soil fertility and interactions with soil bacteria. Measuring the relative importance of nutritional and microbial drivers of herbivore resistance has proven problematic, in part because it is difficult to manipulate soil-bacterial community composition. Here, we exploit variation in soil fertility and microbial biodiversity across 20 farms to untangle suppression of aphids (*Brevicoryne brassicae*) through bottom–up and top–down channels. We planted *Brassica oleracea* plants in soil from each farm, manipulated single and dual infestations of aphids alone or with caterpillars (*Pieris rapae*), and exposed aphids to parasitoid wasps (*Diaeretiella rapae*) in the open field. We then used multi-model inference to identify the strongest soil-based predictors of herbivore growth and parasitism. We found that densities of *Bacillus* spp., a genus known to include plant-growth-promoting rhizobacteria, negatively correlated with aphid suppression by specialist parasitoids. Aphid parasitism also was disrupted on plants that had caterpillar damage, compared to plants attacked only by aphids. Relative abundance of *Pseudomonas* spp. bacteria correlated with higher aphid growth, although this appeared to be a direct effect, as aphid parasitism was not associated with this group of bacteria. Non-pathogenic soil bacteria are often shown to deliver benefits to plants, improving plant nutrition and the deployment of anti-herbivore defenses. However, our results suggest that these plant growth-promoting bacteria may also indirectly weaken top–down aphid suppression by parasitoids and directly improve aphid performance. Against a background of varying soil fertility, microbial biodiversity, competing herbivores, and natural enemies, we found that effects of non-pathogenic soil microbes on aphid growth outweighed those of nutritional factors. Therefore, predictions about the strength of plant defenses along resource gradients must be expanded to include microbial associates.

## Introduction

Chemical defenses induced by plants in response to herbivore attack often trade-off with plant growth due to resource allocation costs, genetic costs, and opportunity costs of prioritizing defense-associated physiological processes over growth (Züst and Agrawal, 2017). Growth/defense investments are traditionally discussed along a continuum of resource availability, where investments in defense diminish under high nutrient conditions (Coley et al., 1985; Herms and Mattson, 1992). Defense induction is also governed by herbivore community composition (Stam et al., 2014) and microbial associates of host plants (Pineda et al., 2017), among numerous other ecological factors. For example, production of secondary metabolites induced by chewing herbivores can be constrained when chewers and suckers co-occur, due to antagonism between defense signaling pathways (Thaler et al., 2012), while plant-growth-promoting rhizobacteria (PGPR) can prime inducible defenses in advance of herbivory (Pineda et al., 2010). *Pseudomonas simiae* (formerly known as *P. fluorescens*), a well-known PGPR, increases resistance to chewing herbivores (Pangesti et al., 2015a), but has been found to interfere with volatile-mediated attraction to parasitoids of sucking herbivores (Pineda et al., 2013). Indeed, complex interactions between soil microbes, plants, herbivores, and natural enemies suggest that simple predictions made about defense investments along resource gradients are inadequate, but the importance of each of these numerous drivers of herbivore defense remain unclear.

While rapid advances have been made in understanding how microbe-mediated plant defenses influence multi-trophic interactions, knowledge has largely emerged from experiments with single-strain inoculations of defense-priming bacterial taxa (Pangesti et al., 2013; Pineda et al., 2017). Manipulating multiple bacterial taxa is a considerable challenge, but efforts to move from examining single PGPR species to whole bacterial and fungal communities suggest that rhizobiome diversity may reduce the vulnerability of plants to herbivore attack (Hol et al., 2010). However, effects of naturally diverse soil microbes on plant defenses in multi-herbivore communities have yet to be elucidated. Interactions between diverse microbes, plants and diverse herbivores are likely to be complex because microbe-mediated defense can act on herbivores directly, via production of secondary metabolites, and also indirectly, via production of herbivore-induced plant volatiles that attract natural enemies, such as predators and parasitoids (Pangesti et al., 2013). Examining these microbe-mediated tritrophic interactions in field environments is critical to understanding the function, context-dependence, and utility of PGPRs for plant protection in agricultural systems.

While communities of rhizobacteria remain challenging to manipulate, the recent affordability of next-generation sequencing (NGS) technology enables an observational approach to quantifying effects of the ambient rhizobiome on herbivores. Here, we use 16S sequencing to describe the diversity and relative abundance of soil bacteria across a gradient of soil fertility using soil collected from 20 organic farms, evaluating the predictive strength of microbial and fertility-based predictors of herbivore growth and suppression by natural enemies in the field. Then we examine effects of single and dual infestations of chewing and sucking insects on herbivore growth on *Brassica oleracea* plants along this same gradient in soil quality. We use this correlative approach to examine the relative importance of microbial symbionts and resource availability in top–down and bottom–up suppression of herbivores in diverse field environments.

## Materials and Methods

### Natural History

At our field site, broccoli plants (*Brassica oleracea* var. capitata, cvs. Arcadia) are attacked by herbivorous aphids and caterpillars, while the aphids (but generally not the caterpillars) are in turn attacked by parasitoid wasps (Snyder et al., 2006; Blubaugh et al., 2018). The cabbage aphid (*Brevicoryne brassicae*) is the dominant aphid on these plants, while *Pieris rapae* is the most common caterpillar species. The most abundant aphid parasitoid in the system is *Diaeretiella rapae*, which limits cabbage aphid population growth in the U.S. Pacific Northwest (Blubaugh et al., 2018); however, parasitism of *P. rapae* is rare in the region (Blubaugh et al., 2018). Caterpillars (*P. rapae*) used in our experiments came from a lab colony, and aphids (*B. brassicae*) came from a wild colony collected from a single kale plant (*Brassica oleracea* var. acephala) on April 12, 2016.

### Soil Collection

**FIGURE 1:**
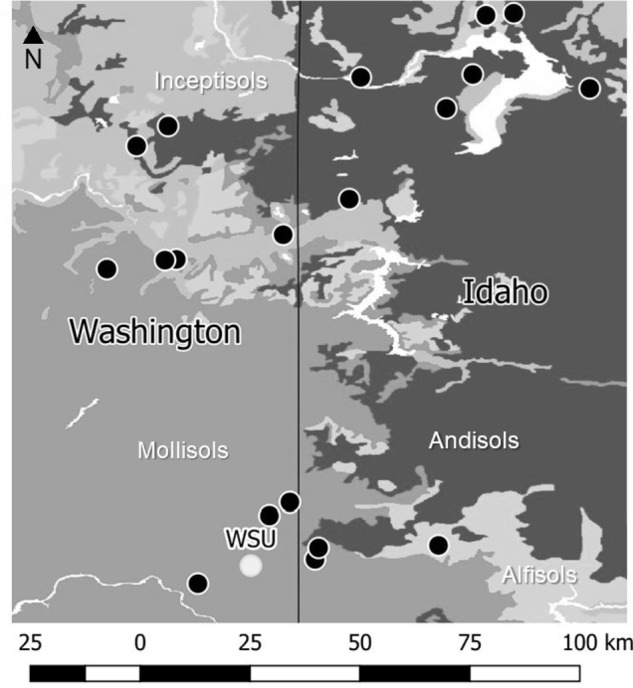
Map of 20 sites and underlying soil orders in Eastern Washington and Northern Idaho, United States, where soil was collected from collaborating organic vegetable farms for a common garden experiment. The experiment took place at the Eggert Family Organic Farm at Washington State University, indicated in white.

On April 20, 2016, 19 L of soil from the 1–15 cm profile were collected from 20 organic mixed-vegetable farms in eastern Washington and northern Idaho (**Figure 1**). Map was composed in QGIS (QGIS Development Team, 2018), using data from the USA Web Soil Survey (Soil Survey Staff et al., 2018). Soil cores (10-cm diameter) were collected every 1 m in beds designated for *Brassica* plantings by our cooperating growers, timed to synchronize with growers’ bed preparation for transplanting. Because microbial communities change rapidly following soil disturbance events (Doran, 1980), all samples included in our study were collected in a single day and immediately deposited in cold storage, and seedlings were transplanted into it the following morning. Unfortunately, the necessity of sampling in a single day limited the number of sites (20) and the number of replicates within sites (2) that were possible for our team to acquire for this study. Soil samples from each farm were sent to Soiltest Farm Consultants (Moses Lake, WA, United States) immediately following collection. There, soil samples were passed through a 2-mm sieve and analyzed for the following properties according to recommended soil-testing methods by Gavlak et al. (2003): nitrate-nitrogen (N) using the chromotropic acid method; ammonium-N with the salicylate method; Olsen phosphorus; NH_4_OAc extractable potassium; DTPA-Sorpitol extractable sulfur; soil pH in a 1:1 w/v water saturated paste; and percent soil organic matter by loss on ignition method. Microbial biomass was estimated using a Solvita test (Haney et al., 2008). The remaining soil was stored overnight at 4 degrees C before planting in the greenhouse (described next).

### Aphid Population Growth and Parasitism in the Field

To evaluate the effects of soil fertility and soil bacteria on aphid growth and parasitism on plants with or without caterpillar herbivory, we measured aphid colony growth and parasitism across all 20 farm soils in a common garden at Eggert Family Organic Farm at Washington State University in Pullman, WA, United States. The morning after soil samples were collected (April 21), broccoli seedlings (*B. oleracea* var. *Capitata* svs. *Arcadia*) were transplanted into 2.8-L pots of soil collected from each farm (2 pots × 20 replicate farms = 40 plants), and completely randomized in the greenhouse. After growing in farm soils for 5 weeks in the greenhouse on 16/8 h light/dark cycles at 26 degrees C, pots were completely randomized and placed in the field on June 1, 2016. Twenty-five aphids were placed on all plants, and 2nd instar *P. rapae* caterpillars were added to one plant from each farm soil treatment (2 caterpillar treatments × 20 replicate farms = 40 total replicates). Aphids were counted every 96 h and after 2 weeks (the amount of time required for parasitoid development), proportions of aphids parasitized were estimated by counting aphid mummies (mummies are hardened pupal cases left behind after parasitoids emerge from killed aphids). For the first week, the mixed-age aphid colonies were controlled to keep aphid densities relatively similar (within 50 aphids/plant) by gently brushing aphids off plants with colonies that grew more quickly than others.

### Soil Bacterial Community Profiling

The soil microbial community was destructively sampled from an extra control plant (with no herbivore damage) from each farm soil replicate on June 8, 2016 by gently shaking roots loose from the soil, and then shaking soil from the fine root region through a 2-mm mesh sieve to remove small root parts. Microbial DNA from two 0.25-g subsamples was extracted immediately following using MoBio soil extraction kits (Qiagen, Inc., Germantown, MD, United States).

The bacterial amplicon library was generated using PCR primers 341F/785R (Klindworth et al., 2013), with Illumina adapter overhang sequences, to target the V3–V4 hypervariable regions (∼464 bp) of the 16S rRNA gene. The library was prepared using a two-step PCR protocol, following the Illumina Metagenomic Library Prep Guide^1^ and Nextera XT index kit (Illumina, Inc., San Diego, CA, United States) for sample multiplexing. PCR products were cleaned with an Agencourt AMPure XP kit (Beckman Coulter, Brea, CA, United States), quantified with a Quant-IT HS-DNA dsDNA assay (Thermo Fisher Scientific, Inc., Waltham, MA, United States), then normalized and pooled at equimolar concentration. The pooled library was then sequenced on an Illumina MiSeq at the Center for Genome Research and Biocomputing (Oregon State University, Corvallis, OR, United States) using 2 bp × 300 bp paired-end V3 chemistry. Raw sequences are available on the NCBI Sequence Read Archive (SRA) under study accession SRP152350.

Demultiplexed sequences were initially trimmed of trailing low-quality bases and then merged using Trimmomatic v.0.36 (Bolger et al., 2014) with the following settings: LEADING = 3, TRAILING = 3, HEADCROP = 15, SLIDING WINDOW = 5:15 and MINLEN = 100. Merged reads were then fed into the *DADA2* pipeline (v.1.6.0; Callahan et al., 2016) in R (v. 3.4.0; R Core Development Team), error-corrected, and assembled into amplicon sequence variants (ASVs). ASVs have many benefits over traditional operational taxonomic unit (OTU) methods, such as revealing cryptic diversity through identification of exact biological sequences that differ by a single nucleotide, among others (Callahan et al., 2017). Once assembled, chimeras were detected, removed, and taxonomic information was then assigned to each ASV using the RDP Naïve Bayesian Classifier (Wang et al., 2007), trained to the RDP training set (v.14). ASV read counts were then averaged across the two subsamples for each farm, and those that failed to classify to kingdom or were identified as chloroplast or mitochondrial sequences, respectively, were discarded. Moreover, singletons, as well as rare, low abundance taxa were also filtered from the dataset prior to analyses, with ASVs with a minimum read count of 5 and occurring in at least 10% of our samples being retained. Post-filtering, *DADA2* inferred 4,621 unique, error-corrected ASVs representing 1,294,852 sequences, on which analyses below were performed.

Post-processing, ASV richness and diversity (Shannon index) were calculated at the farm level using the *phyloseq* package (McMurdie and Holmes, 2013). Calculations were performed on rarefied data (39185 reads per farm). These metrics were also used to estimate microbial community evenness, following Pielou’s index (Pielou, 1969). Finally, as we were interested in the role of known PGPRs in mediating aboveground tritrophic interactions, we also calculated the relative abundance of *Bacillus* and *Pseudomonas* spp. (ASVs agglomerated at the genus level) observed at each farm.

### Analyses

All analyses were performed in R (v. 3.4.0; R Core Development Team), and data used in analyses are available in **Supplementary Table S1**. To examine herbivore resistance across farms, we applied an information theoretic approach to identify the strongest uncorrelated soil-based predictors of herbivore performance and rates of parasitism. Prior to running global models, regressions of all potential pairs of predictor variables were plotted in R using the *pairs* function, and only one variable from collinear pairs was selected for inclusion in the global model. Our first model used the *lme* function in the *nlme* package (Pinheiro et al., 2014) to predict parasitism of *B. brassicae*, and the global model contained the following variables: concentrations of nitrate-N, ammonium-N, plant available P, K, SO_4,_ pH, organic matter, *Bacillus* spp. relative abundance, *Pseudomonas* spp. relative abundance, microbial community evenness, and microbial biomass (mg/kg soil). We selected our variables and hypotheses for the global model based on known relationships between soil nutrient pools, soil microbes, host plant quality, and herbivore defenses (**Table 1**). To simplify models, we analyzed means of proportions of aphids parasitized across the five sample dates, and square root transformed them to meet normality and variance assumptions. We included ‘farm’ as a random intercept because the model included two replicates from each of the 20 farms (both herbivory treatments were included). One outlier replicate plant (from the 40 replicates) was removed from our models because its aphid colony count was more than 3 standard deviations higher than the mean of the whole group, and twice as high as the next largest colony. We expect that such rapid growth was due to confounding factors unrelated to plant quality (i.e., immigration from adjacent areas). Competing models were evaluated using the *dredge* function in the *Mumin* package (Bartoń, 2014), and best-fit models were chosen by selecting the model that included the fewest variables within two corrected Akaike information criterion (AICc) values of the minimum (Burnham and Anderson, 2004). Because we experimentally manipulated caterpillar co-herbivory, we did not include this variable in the correlative model selection process. Instead, we ran a separate model to more robustly test effects of our herbivory treatments on aphid parasitism across the 20 farms. Two replicates were removed from the caterpillar model because the caterpillars died on these plants without consuming any leaf tissue.

**Table 1:** Hypotheses and associated evidence for each of the soil-based predictors of aphid growth and parasitism examined in our global models.

Factor	Hypothesis	Reference
Soil NO_3_, Soil NH_4_, P, K, SO_4_	N, P, K, and S availability increases aphid growth and parasitism	Coley et al., 1985; Price, 1991
pH	Acidic soils decrease aphid growth and decrease parasitism	Birkhofer et al., 2008
Organic matter	Organic matter decreases aphid growth and increases parasitism	Altieri and Nicholls, 2003
Microbe biomass	Microbial activity decreases aphid growth and increases parasitism	Birkhofer et al., 2008
*Bacillus* spp.	*Bacillus* spp. decrease aphid growth and increase parasitism.	Gadhave and Gange, 2016; Gadhave et al., 2016a
*Pseudomonas* spp.	*Pseudomonas* spp. increase aphid growth and decrease parasitism	Pineda et al., 2012, 2013

To examine effects of the same soil-based predictor variables on aphid colony growth, we performed another model predicting mean aphid counts, pooled across seven sample dates. Random effect structure and predictors in the global model were the same as described above for the model predicting aphid parasitism, and the best-fit subset of predictors was identified in a similar manner. A separate model tested the effects of caterpillar co-herbivory on aphid growth. All scatterplots were made using function *visreg* in the *visreg* package (Breheny and Burchett, 2016) and were fit using output from best-fit models.

## Results

### Soil Microbial Gradients

**FIGURE 2:**
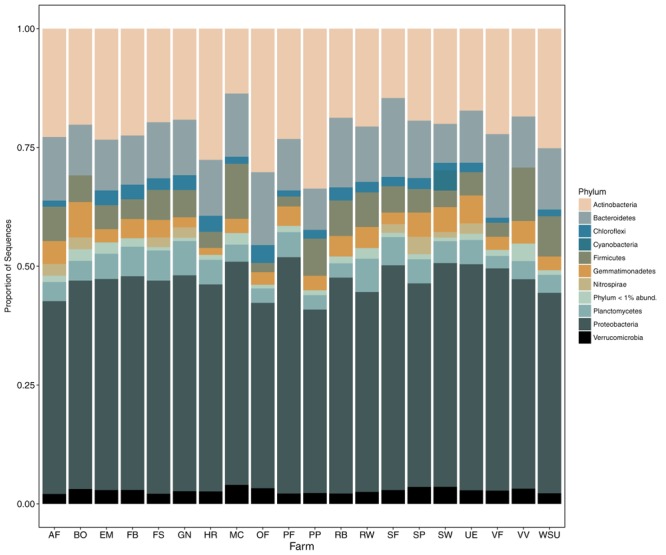
Relative abundance (proportion of sequences) of the 10 most common soil bacterial phyla across the 20 farms surveyed.

A total of 18 bacterial phyla were found, of which *Proteobacteria* was most dominant, ranging from 38.6 to 49.7% of total sequences across farms sampled (**Figure 2**). Other abundant phyla observed included *Actinobacteria* (range: 13.6–33.6%), *Bacteroidetes* (range: 8.2–17.6%), *Firmicutes* (range: 1.9–11.6%), *Planctomycetes* (range: 2.6–7.2%), and *Gemmatimonadetes* (range: 1.5–7.5%). We identified known PGPR *P. fluorescens* at the species level, while the majority of other *Pseudomonas* ASVs failed to match beyond the genus level. *Pseudomonas* spp. include both PGPR and pathogenic species (Haney et al., 2017); however, we found little/no evidence of pathogenic *P. syringae* presence in our sequences during ASV-level analyses. *Bacillus* taxa identified at the species level, included *B. coagulans, B. lentus, B. drentensis*, and *B. acidiceler.* Because none of the well-known pathogenic species were identified in our samples, our subsequent analyses included relative abundances of *Bacillus* spp. and *Pseudomonas* spp. ASVs agglomerated at the genus level.

### Aphid Colony Growth and Parasitism

**FIGURE 3:**
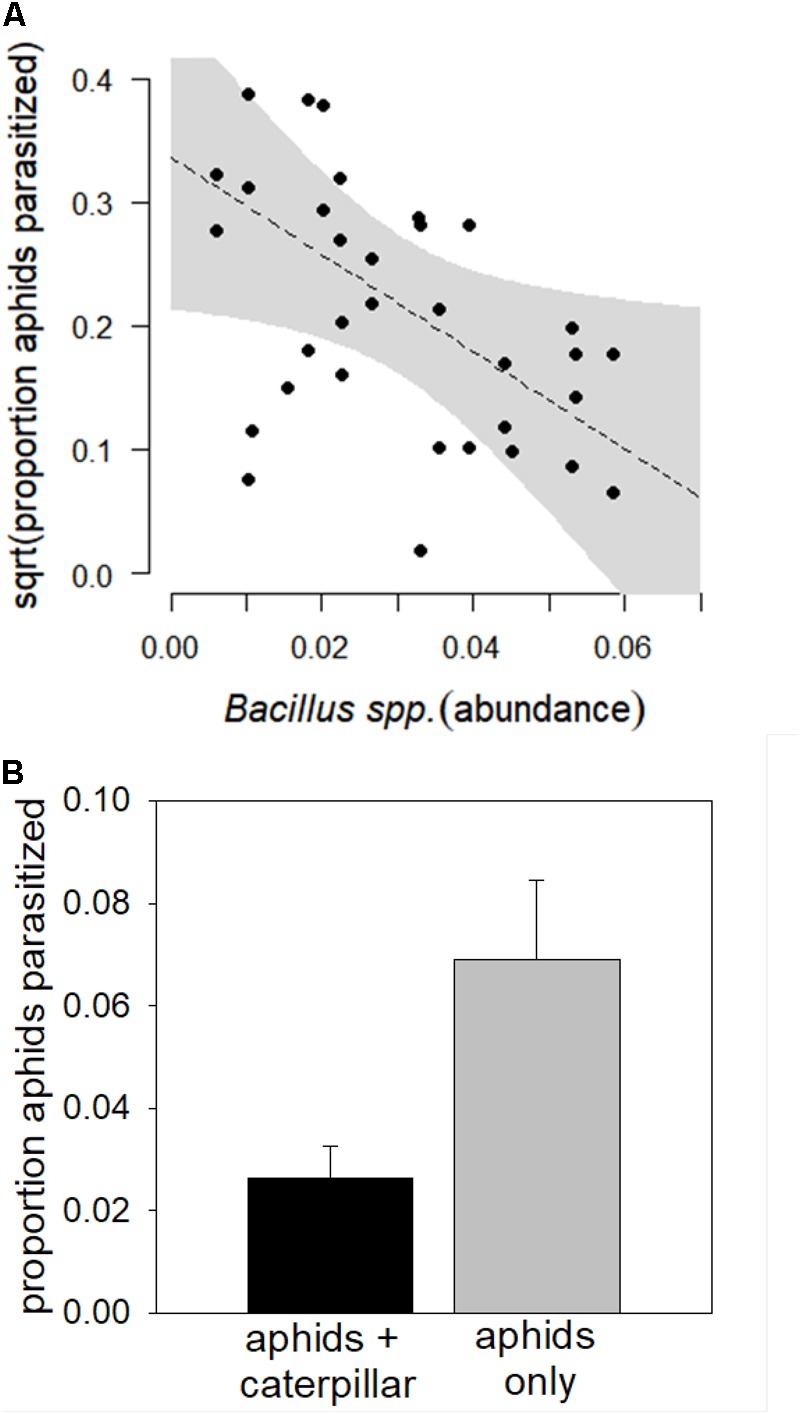
Relationship between **(A)** *Bacillus* spp. relative abundance and **(B)** caterpillar co-herbivory and parasitism of cabbage aphids (*B. brassicae*) in a common garden field assay across 20 organic farm soils. Proportions of aphids parasitized were square root transformed prior to analysis to meet model assumptions; the scatterplot represents output from the mixed-effect model and the shaded gray area indicates a 95% confidence interval. Error bars indicate standard error of the mean.

The top model predicting aphid parasitism contained only *Bacillus* spp. (**Figure 3A** and **Table 2**; marginal *R*^2^ = 0.0928, conditional *R*^2^ = 0.113). Relative abundance of *Bacillus* marginally negatively correlated with aphid parasitism (Coefficient = -1.445, *SE* = 1.277, T = -1.974, *P* = 0.0639). Across diverse soil communities, caterpillar co-herbivory reduced aphid parasitism by 57% (**Figure 3B**, Coefficient = 0.089, *SE* = 0.036, T = 2.461, *P* = 0.0242).

**Table 2:** Model selection table for the top soil-based predictors of aphid parasitism across 20 organically managed farm soils (*Bacillus* spp. relative abundance, *Pseudomonas* spp. *relative abundance*, bacterial community evenness).

Model	Intercept	*Bacillus* spp.	*Pseudomonas* spp.	Bacterial community evenness	AICc	Delta	Weight
1	0.2640	–2.48	0.3489		–42.5	0	0.297
2	0.5705	–2.441	0.698	–0.342	–42.0	0.49	0.232
3	0.2660	–2.458			–40.6	1.85	0.118
4	0.5002	–2.409		–0.2595	–40.1	2.36	0.09
5	0.2024		–0.9867		–39.6	2.93	0.068
6	0.7055		–0.3513	–0.5599	–39.4	3.06	0.064
7	0.1945				–37.4	5.07	0.026
8	0.7278			–0.5876	–37.3	5.16	0.022

*Delta indicates the difference in AICc from the best-fit model, and the AICc weight of each model, which represents the likelihood of the model relative to competing ones. Retained top model is shaded gray, and factors that were not retained in any of the top eight candidate models were eliminated from tables. Original global model included the following factors: concentrations of nitrate-N, ammonium-N, plant available P, K, SO_*4*,_ pH, organic matter, *Bacillus* spp. relative abundance, *Pseudomonas* spp. relative abundance, microbial community evenness, and microbial biomass (mg/kg soil).*

**FIGURE 4:**
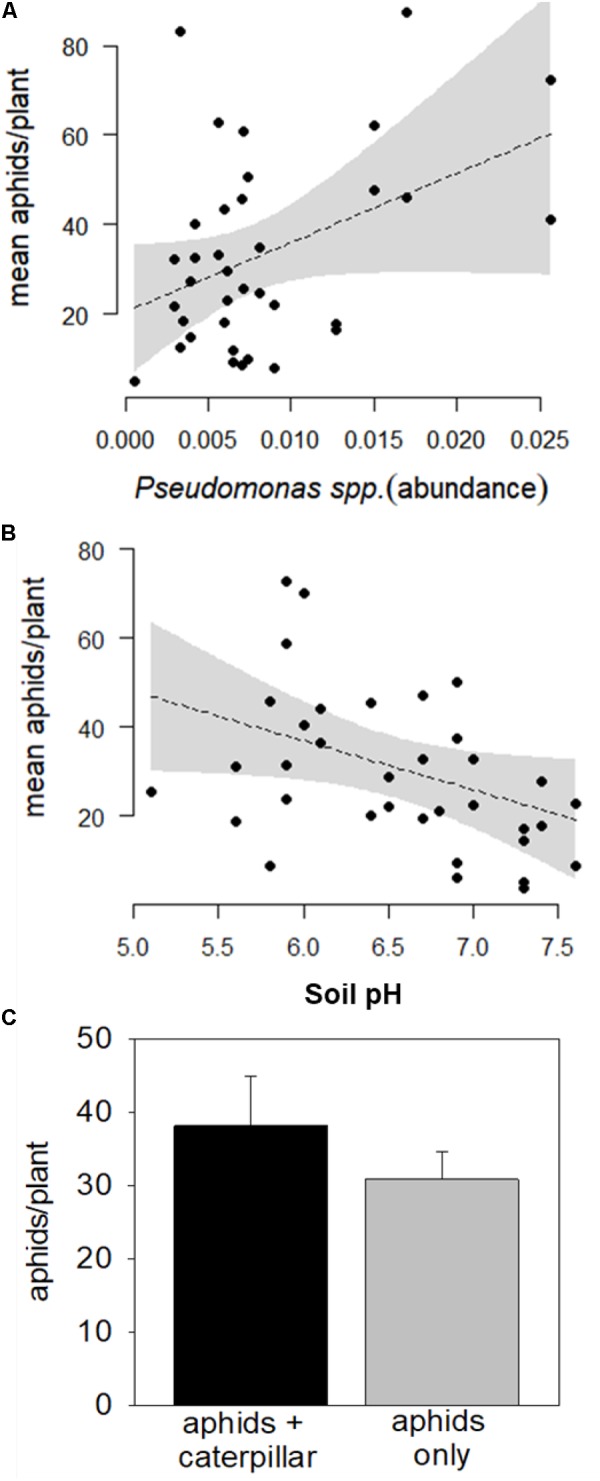
Relationship between **(A)** *Pseudomonas* spp. relative abundance, **(B)** soil pH, and **(C)** caterpillar co-herbivory on colony growth of cabbage aphids (*B. brassicae*) in the common garden field assay across 20 organic farm soils. Scatterplots represent output from mixed-effects models and the shaded gray area indicates a 95% confidence interval. Error bars indicate standard error of the mean.

The top model predicting aphid colony growth contained *Bacillus* spp., *Pseudomonas* spp., bacterial community evenness, and pH (**Table 3**; conditional *R*^2^ = 0.2177957, marginal *R*^2^ = 0.272799). Of these, only *Pseudomonas* spp. and pH significantly correlated with aphid growth. Aphid colony growth increased with *Pseudomonas* spp. relative abundance in the soil, and marginally decreased with increasing soil pH (**Figures 4A,B** and **Table 4**). Despite the strong negative effect of caterpillar co-herbivory on aphid parasitism, caterpillars did not significantly increase aphid colony growth (**Figure 4C**, Coefficient = -6.27325, *SE* = 6.837, T = -0.917, *P* = 0.372).

**Table 3:** Model selection table for the top soil-based predictors of aphid growth across 20 organically managed farm soils (*Bacillus* spp. relative abundance, *Pseudomonas* spp. relative abundance, bacterial community evenness, NH_4_, percent organic matter, and pH).

Model	(Intercept)	*Bacillus* spp.	*Pseudomonas* spp.	Bacterial community evenness	NH_4_	% Organic matter	pH	AICc	Delta	Weight
1	191.60	72.28	1667	–127.7			–11.06	276.3	0	0.263
2	178.30	77.66	1772	–116		1.365	–11.6	276.4	0.13	0.246
3	194.60	71.78	1834	–129.4	–0.21		–11.53	278.4	2.16	0.089
4	178.50	77.62	1780	–116.1	–0.01	1.36	–11.63	278.8	2.57	0.073
5	20.25		1709					333.6	26.13	0
7	29.92	133.60						342.2	34.75	0
8	9.619			26.67				342.9	35.43	0
9	73.45						–6.11	349	41.48	0
10	33.82							352.9	45.45	0

*Delta indicates the difference in AICc from the best-fit model, and AICc weights represent the likelihood of the model relative to competing ones. Retained best-fit model is shaded gray, and factors that were not retained in any of the top five candidate models were eliminated from tables. Models 5–10 are single-parameter and intercept-only models for reference. Original global model included the following factors: concentrations of nitrate-N, ammonium-N, plant available P, K, SO_*4*,_ pH, organic matter, *Bacillus spp.* relative abundance, *Pseudomonas* spp. relative abundance, microbial community evenness, and microbial biomass (mg/kg soil).*

**Table 4:** Output from the top model predicting aphid colony growth over 7 weeks across 20 organically managed farm soils.

Predictor	Coefficient	*SE*	*T*	*P*-value	
(Intercept)	159.51	165.76	0.96	0.348	
pH	–9.95	5.14	–1.93	0.0723	.
*Pseudomonas* spp.	2046.47	608.92	3.36	0.0043	^∗^
*Bacillus* spp.	95.44	210.46	0.45	0.6567	
Bacterial community evenness	–88.41	191.06	–0.46	0.6502	

*^∗^Denotes significance at the 0.05 level of alpha.*

## Discussion

We provide new evidence of the importance of plant–microbe associations in tritrophic interactions by examining them in naturally diverse soil communities across a gradient of resource availability. Here, we trade experimental control for ecological relevance by evaluating numerous correlative predictors of herbivore performance across 20 different farm soils and their unique bacterial communities in a common garden. In contrast to experiments using inoculations of isolated PGPR strains (Zehnder et al., 1997; Gadhave and Gange, 2016), we found no evidence of herbivore suppression associated with either *Bacillus* spp. or *Pseudomonas* spp. relative abundance in the ambient soil microbial community. Rather, our data suggest that these bacterial genera may increase susceptibility of *B. oleracea* to aphids in field environments. None of the soil nutrients we examined associated with aphid growth. This suggests that plant–microbe interactions might have stronger effects on cabbage aphids than nutrient limitation.

*Bacillus* spp. negatively associated with aphid parasitism rates (**Figure 3A**). This pattern from soil communities collected from the field is consistent with another controlled laboratory experiment showing that PGPRs can interfere with volatile signaling that enables parasitoids to locate prey (Pineda et al., 2013). While *Bacillus* spp. are effective at priming chemical defenses induced by the jasmonic acid (JA) and ethylene defense signaling pathways (Pozo et al., 2008; Pangesti et al., 2013), as well as increasing parasitoid attraction to caterpillars (Pangesti et al., 2015b), they have also been found to induce susceptibility to phloem-feeding insects (Shavit et al., 2013). In contrast, other studies found that single-strain inoculations of different *Bacillus* strains reduced aphid growth (Valenzuela-Soto et al., 2010) and parasitism (Gadhave et al., 2016a); however, negative effects may be neutralized in mixed-strain treatments (Herman et al., 2008; Gadhave et al., 2016a,b) that more accurately reflect the diverse microbial communities of organically managed soils we examine here (Li et al., 2017; Schmid et al., 2017). We suspect that one or more *Bacillus* taxa, including *B. coagulans, B. lentus, B. drentensis*, and *B. acidiceler* identified in our study, may have interfered with parasitoid attraction.

Co-infestation with caterpillars also reduced aphid parasitism (**Figure 3B**). A competing chewing herbivore might dilute or constrain volatile signals exploited by aphid parasitoids to find their prey (Vos et al., 2001), reducing top–down suppression. Indeed, our recent fieldwork in this system showed that co-herbivory by chewing herbivores reduced aphid parasitism as well as concentrations of secondary metabolites important in aphid-specific volatile signaling (Blubaugh et al., 2018). This consistent pattern detected across a gradient of microbial diversity further emphasizes the importance of herbivore community structure in determining the outcome of tritrophic interactions.

The best-fit model predicting aphid colony growth contained *Pseudomonas* spp., which associated with greater aphid increase (**Figure 4A**), and soil pH, which marginally associated with decreased aphid colony growth (**Figure 4B**). Again, while *Pseudomonas* PGPRs have often been found to induce systemic resistance to chewing herbivores (Pineda et al., 2010; Hol et al., 2013), our results suggest that these well-known PGPRs may have facilitated growth of phloem feeders (e.g., Pineda et al., 2012). This putative case of induced susceptibility could have occurred because of a tradeoff in defense signaling pathways, as *Pseudomonas* may suppress salicylic acid-mediated defenses by priming induction of the JA pathway (e.g., Haney et al., 2017). However, defenses induced along the JA pathway are more likely to limit aphid growth rather than facilitate it Züst and Agrawal (2016). Instead, cabbage aphid growth may correlate with the relative abundance of JA-priming PGPRs in our study because specialist cabbage aphids (*B. brassicae*) use secondary metabolites as feeding stimulants and are often not limited by them (**Figure 3C**, Züst and Agrawal, 2016). Indeed, *B. brassicae* densities can increase in response to high glucosinolate concentration (Cole, 1997; Stafford et al., 2012), as they selectively sequester glucosinolates for their own defenses against predators (Kazana et al., 2007; Kos et al., 2012). For this reason, cabbage aphids notoriously perform better in organically managed soils (Staley et al., 2010; Stafford et al., 2012), which facilitate chemical defense induction, compared with conventionally managed soils that generally have lower levels of biological activity (Mäder et al., 2002).

The marginally negative effect of higher soil pH on cabbage aphid growth in our study (**Figure 3B**) may relate to historical soil inputs at our sites. Soil acidification can be a legacy of long-term ammoniacal fertilizer inputs in intensified conventional farming systems (Barak et al., 1997; Birkhofer et al., 2008), and many of our participating organic farms recently transitioned from such systems. Acidic soils can reduce plant vigor and may constrain anti-herbivore defenses (e.g., Birkhofer et al., 2008). Generally, the marginal effects of soil pH on aphid growth, along with other non-significant factors associated with soil fertility and host plant quality, suggest that microbial symbionts in our system (*Pseudomonas* spp. and *Bacillus* spp.) may be stronger drivers of herbivore performance than the nutritional components of chemical defense. Although the expense and challenge of collecting and sequencing soils from numerous sites limited the replication of our study, this work is biologically meaningful because there is still a dearth of evidence from the field describing microbe-mediated interactions (Schreiter et al., 2018). As the affordability of microbial community sequencing improves, it is critical to evaluate tritrophic interactions in their realistic ecological contexts, accounting for diversity in microbial and herbivore community structure.

These results suggest a pattern of induced susceptibility to aphids by bacterial taxa that are usually considered PGPRs, ostensibly via direct and indirect (volatile and parasitoid-mediated) pathways. Because a correlative approach is necessary for evaluating ambient bacterial communities in the soil, we cannot determine causal links or provide clear mechanistic detail. However, our results from the field are consistent with experimental greenhouse work showing PGPR-induced susceptibility to phloem feeders (e.g., Pineda et al., 2012, 2013). This first step at evaluating the function and importance of known PGPRs in soil communities implies that there are costs as well as benefits of plant-colonizing soil microbes that prime plant defensive processes, and that these costs likely depend on the identity of herbivores (i.e., chewer or phloem-feeder) and the intensity of herbivore pressure. Next steps will (1) link these patterns in relative abundance of PGPRs with defensive plant chemistry, (2) identify environmental drivers of soil bacterial community structure, such as soil disturbance or fertility amendment materials, and 3) characterize interaction webs between naturally occurring soil microbes, naturally diverse herbivore communities, and volatile-mediated prey suppression. Importantly, neither herbivore growth nor top–down suppression associated with soil fertility in this study; this suggests that predictions made about plant investments in chemical defense based merely on resource availability are inadequate to describe the complexity of plant-mediated interactions in multi-trophic, real-world communities.

## Author Contributions

CB, WS, JR, and LC-B designed the experiment. CB performed experiment and wrote the paper. RS performed bioinformatics. All co-authors edited drafts of the manuscript.

## Conflict of Interest Statement

The authors declare that the research was conducted in the absence of any commercial or financial relationships that could be construed as a potential conflict of interest.

## Footnotes

1 https://support.illumina.com/content/dam/illumina-support/documents/documentation/chemistry_documentation/16s/16s-metagenomic-library-prepguide-15044223-b.pdf

## Supplementary Material

TABLE S1 Soil nutrient and microbial data, insect counts on broccoli plants, and site locations where soil samples were collected in April 2016 for a common garden experiment.
